# Unexpectedly prolonged fasting and its consequences on elderly patients undergoing spinal anesthetics. A prospective observational study^1^

**DOI:** 10.1590/s0102-865020190030000009

**Published:** 2019-03-18

**Authors:** Oguzhan Yeniay, Zeki Tuncel Tekgul, Onur Okur, Noyan Koroglu

**Affiliations:** I MD, Izmir Dr. Suat Seren Chest Diseases and Surgery Training and Research Hospital, Department of Intensive Care, Izmir, Turkey. Conception and design of the study; acquisition, analysis and interpretation of data; technical procedures; manuscript writing; final approval.; II Associate Professor, Izmir Bozyaka Training and Research Hospital, Department of Anesthesiology and Reanimation, İzmir, Turkey. Scientific, intellectual, conception and design of the study; statistics analysis; critical revision; final approval.; III MD, Istanbul Okmeydani Training and Research Hospital, Department of Anesthesiology and Reanimation, Istanbul, Turkey. Conception and design of the study, manuscript preparation and writing, critical revision, final approval.; IV MD, Izmir Katip Celebi University, Ataturk Training and Research Hospital, Department of Anesthesiology and Reanimation, Izmir, Turkey. Conception and design of the study, analysis and interpretation of data, technical procedures, manuscript preparation, final approval.

**Keywords:** Fasting, Anesthesia, Spinal, Myocardial Ischemia, Geriatrics, Orthopedics

## Abstract

**Purpose:**

To measure the preoperative fasting durations with respect to time of the day and its effect on vital parameters and electrocardiogram in elderly patients undergoing surgery under spinal anesthesia.

**Methods:**

This study investigated 211 patients older than 60 years undergoing elective surgery under spinal anesthesia. Patients scheduled for surgery in morning hours (AM) and afternoon hours (PM) were compared. Patients fasting hours and repeated measurements of mean arterial pressure (MAP), heart rate (HR), peripheral oxygen saturation (Sp02) and the type and number of ischemic electrocardiogram (ECG) signs were recorded and compared [preoperative, zeroth, 2nd,5th,15th,30th minutes following spinal anesthesia(SA)].

**Results:**

Mean fasting durations were 12±2.8 and 9.5±2.1 hours in AM group and 15.5±3.4 12.7±4.4 hours in PM group for foods and liquids respectively. ECG changes were significantly more frequent in PM group and body temperatures were significantly higher in AM group patients.

**Conclusion:**

Our study has shown that fasting times in our population is far longer than recommended and fasting prolonged>15 hours is related to a transiently increased cardiac stress and mild hypothermia.

## Introduction

 Halting oral intake in the night before surgery has long been a part of routine preoperative preparation to avoid a possible aspiration of gastric content in lungs. Unconciousness and supression of protective airway reflexes associated with anesthesia and sedation may result in regurgitation and vomiting of gastric content when gastric pressure exceeds lower eosephageal pressure which in turn causes aspiration of gastric content into lungs^1^. 

Recent advancements demonstrate that longer fasting periods provide hazards rather than benefit compared to shorter ones^2^ ^,^ ^3^. Thus, clinical practice seems to have evolved to a much shorter course of fasting. 

Unfortunately, we suspect that these scientific advancements might not reflect to daily clinical practice of the busy training hospitals. Staff shortages, inadequate communicative skills of the staff, vast number of daily cases, fear of legal consequences and economic circumstances in tertiary teaching institutions may cause prolonged preoperative fasting durations. Likewise, it is shown that a shorter period of fasting is rather said than done in many hospitals worldwide^4^ ^-^ ^7^. We believe that patients scheduled for surgery in the later hours of the day might be more clearly exposed to prolonged fasting periods. This prolongation might easily be tolerated by healthy and young patients whereas special populations such as elderly patients might be more susceptible to deleterious effects induced by prolonged fasting. 

We hypotethize that patients scheduled for surgery in the later hours of the day are exposed to longer fasting and this prolonged fasting is related to deleterious effects in the elderly. We believe the most prominent of these effects should be on cardiovascular system. Our primary outcome was the fasting duration between groups and secondary outcomes included number of ischemic changes on electrocardiogram (ECG), MAP, HR, Sp0_2_ and body temperature differences. 

In this setting, we aimed to measure the preoperative fasting durations and investigate the its effects on vital parameters and ECG changes of elderly patients undergoing procedures under spinal anesthesia, which might act as a second stressor on cardiovascular system, further unmasking the deleterious effects of fasting.

## Methods

 This prospective observational study was conducted following approval of the hospital’s human research ethics board and in accordance with Helsinki declaration. This study was registered prospectively to “ClinicalTrials.gov” protocol registry system. 

Two hundred and eleven patients undergoing elective urologic or orthopedics surgery under spinal anesthesia between January and December 2015 were included in this study. Patients older than 60 years of age, classified as Class 1-2 or 3 according to the American Society of Anesthesiologist’s (ASA) Physical Status Classification system were enrolled in this study. Exclusion criteria were; patient refusal to participate, psychiatric disorders, emergency surgery, existing signs of ischemia on ECG and clinical situations mimicking ischemic findings.

Demographics [age, gender, body mass index(BMI)], operation group, existence of comorbidities, operation type and time elapsed since the last oral fluid and food intake until the operation were recorded. Preoperative hydration of every patient with 10ml/kg fluids was administered and hydration amounts were recorded. Patients were observed in two groups according to the time of their operation (the AM group included operations scheduled for between 08.00-12.00 and the PM group included operations scheduled for between 12.00-16.00). Patients’ demographics, vital signs (MAP, HR,Sp0_2_), body temperature]and administered drugs were recorded.

Repeated measurements of MAP, HR, Sp0_2_ and ECG were recorded at certain times (preoperatively, zeroth, 2^nd^, 5^th^, 15^th^ and 30^th^ minutes after SA is placed). Patients’ body temperatures were recorded just before SA is placed. ECG changes were detected via digital analysis specialty of the monitors during routine cardiac monitoring for ischemic signs. Ischemic signs were defined as a negative T wave and ST segment elevation or depression and changes greater than 0.1mm/mV at D2 derivation were recorded. Hyperbaric bupivacaine solution was administered for achievement of SA in all patients. Sensory block levels were determined with loss of cold sensation to alcohol soaked surgical sponge. Dose of the spinal anesthetic administered to patients and sensory block levels were recorded. Patients were administered 2L/min oxygen in operating theater. Hypotension was defined as a decrease in MAP more than 20% following placement of SA and bradycardia was defined as a decrease in HR to lower than 50 bpm following placement of SA. Patients with hypotension were treated with intravenous (iv) ephedrine and patients with bradycardia were treated with 0.5mg iv atropine. Data regarding these adverse events were recorded.

Data analyses were performed with the aid of computer assisted statistics package programme SPSS (Statistical Package for Social Sciences ver.18). Normal distribution of continuous and discrete data were tested with Kolmogorov-Smirnov test. Descriptive variables for continuous and discrete data were expressed as mean±standard deviation (SD) and median while categorical variables were expressed as numbers and percents (%). Non-parametric data were compared using Mann-Whitney U Test, categorical variables were analyzed with Pearson’s Chi-Squared Test or Fisher’s Exact Test. Statistical significance of changes in relation to follow-up times within groups were tested with Wilcoxon Sign-Rank Test.

Repeated clinical measurements were analyzed with Analysis of Variance (ANOVA) for Repeated Measurements Test. Whenever results of the Wilks’ Lambda Test found be statistically significant between multiple groups, Bonferroni Correction for Multiple Comparisons was used to determine the cause of significance between groups. Significance of group vs time interactions was tested with Greenhouse-Geisser Test and whenever the results were found significant percentage changes in repeated measurements were calculated and compared between groups. Unless stated otherwise p values lower than 0.05 were interpreted as statistically significant.

## Results

 Mean age of the patients in the study was 72.5±7.8 and 130 (61.6%) of the patients were male. Mean BMI of the patients were 28.0±4.0. One hundred and thirty-five (62.5%) of the patients had comorbidities with most frequent comorbidity being hypertension. There were no statistically significant differences between groups for age, gender, BMI, ASA class score, comorbidities, operation type, amounts of fluids infused, spinal anesthetic amounts and sensory block levels (p>0.05) (Table 1).

**Table 1:** Comparison of demographics between groups^a^.

		Total	AM	PM	p
Age, Mean±SD (Median)	72.5±7.8 (72)	73.1±7.8 (72)		71.6±8.1 (70)	0.171
Gender	Male, n(%)	130 (61.6)	68 (59.1)	62 (64.6)	0.417
	Female, n(%)	81 (38.4)	47 (40.9)	34 (35.4)	
BMI, Mean±SD (Median)	28.0±4.0(28.1)	28±4.2(28.1)		27.6±3.8 (28.1)	0.600
ASA	ASA II, n(%)	135 (64)	78 (67.8)	57 (59.4)	0.203
	ASA III, n(%)	76 (36)	37 (32.2)	39 (40.6)	
Comorbidity	None, n(%)	58 (27.5)	28 (24.3)	30 (31.3)	0.282
	HT, n(%)	45 (21.3)	34 (29.6)	11 (11.5)	
	DM, n(%)	6 (2.8)	1 (0.9)	5 (5.2)	
	IHD, n(%)	13 (6.2)	8 (7)	5 (5.2)	
	COPD, n(%)	9 (4.3)	4 (3.5)	5 (5.2)	
	CHF, n(%)	3 (1.4)	2 (1.7)	1 (1)	
	Multiple, n(%)	77 (36.5)	38 (33)	39 (40.6)	
Operation type	THR, n(%)	51 (24.2)	30 (26.1)	21 (21.9)	0.135
	TKR, n(%)	47 (22.3)	32 (27.8)	15 (15.6)	
	TUR-BT, n(%)	58 (27.5)	29 (25.2)	29 (30.2)	
	TUP-P, n(%)	33 (15.6)	14 (12.2)	19 (19.8)	
	URS, n(%)	22 (10.4)	10 (8.7)	12 (12.5)	
Hydration amount (ml)	486.3±290.6 (300)	490.9±290.9 (300)		480.7±291.7 (300)	0.812
Amount of spinal anesthetic (ml)	2.4±0.5 (2.5)	2.4±0.5(2.5)		2.4±0.4 (2.5)	0.835
Level of sensorial blockade	T6, n(%)	52 (24.6)	30 (26.1)	22 (22.9)	0.239
	T7, n(%)	24 (11.5)	13 (11.3)	11 (11.5)	
	T8, n(%)	57 (27.0)	28 (24.3)	29 (30.2)	
	T9, n(%)	33 (15.6)	14 (12.2)	19 (19.8)	
	T10, n(%)	45 (21.3)	30 (26.1)	15 (15.6)	

a. (HT: Hypertension, DM: Diabetes mellitus, IHD: Ischemic heart disease, COPD: Chronic obstructive pulmonary disease, CHF: Congestive Heart Failure ,THR: Total hip replacement surgery, TKR: Total knee replacement surgery, TUR-BT: Transuretheral resection of bladder tumours, TUR-P: Transuretheral resection of the prostate, BMI: body mass index, URS: Ureterorenoscopy for ureterolithiasis).

Mean fasting time was 11.0±3.7 hours for fluids and 13.6±3.5 hours for foods. Patients in the PM group had significantly longer fasting times (p<0.001, p<0.001). Mean fasting durations were 12±2.8 and 9.5±2.1 hours in AM group and 15.5±3.4 12.7±4.4 hours in PM group for foods and liquids respectively .Mean operation duration was 86.0±37.4 minutes and there were no statistically significant difference between groups (p>0.05) 

Mean MAP of the patients in the AM group was 98.0±15.1mmHg and 95.1±13mmHg in the PM group patients. There were no statistically significant difference between two groups for preoperative MAP values. (p>0.05).

**Figure 1:**
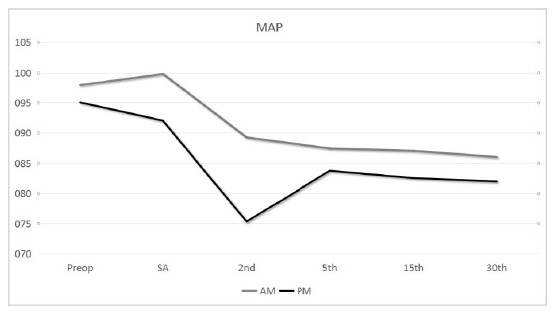
Graphical demonstration for comparison of MAP values versus time between groups.

We found that the patients in the PM group suffered significant decrease in MAP at 2^nd^ minute measurements and change in MAP by time in the PM group was found to be statistically significant (p<0.05) (Fig. 1).

There were no statistically significant differences between groups for MAP changes at the 5^th^, the 15^th^ and the 30^th^ minutes following the spinal anesthetics with regard to preoperative MAP(p>0,05). Nonetheless, there was a statistically significant difference in decrease of MAP values at the 2^nd^ minute (both with regard to preoperative measurement and measurements following spinal anesthetics) in the PM group compared to the AM group (p<0.001, p<0.001 respectively). Increase in MAP values at 5^th^ minute in comparison to 2^nd^ minute was significantly more in PM group than that of AM group. There were no other statistically significant changes in MAP values at any measurement times between groups (p>0.05) (Table 2).

**Table 2:** Comparison of changes in percentage of MAP values between groups^a^.

	AM Mean Change (%)(Min;Max)	PM Mean Change (%)(Min;Max)	P
SA-preop	3.2 (-30.1; 61.6)	-2.5 (-23.5; 25.8)	0,062
2ndmin-preop	-8.8 (-38.4; 11.5)	-20.3 (-51; 1.9)	<0.001
5thmin-preop	-10.6 (-93.2; 9)	-11.5 (-49.1; 4.8)	0.629
15thmin-preop	-10.8 (-33.3; 12.8)	-12.8 (-37.6; 2.1)	0.407
30thmin -preop	-11.9 (-34.8; 15.4)	-13.4 (-40.2; 0)	0.772
2^nd^min- SA	-9.9 (-54.5; 20)	-17.7 (-53.5; 14.6)	<0.001
5^th^min - 2^nd^ min	-0.6 (-22.7; 44.6)	14.2 (-42.2; 100)	<0.001
15^th^ min- 5^th^ min	-0.8 (-22.3; 31.8)	-0.9 (-27.2; 37)	0.561
30^th^ min -15^th^ min	-1.1 (-24.4; 23.3)	-0.6 (-11.2; 23.4)	0.522

a. SA: right after spinal anesthesia, min: minutes.

**Figure 2:**
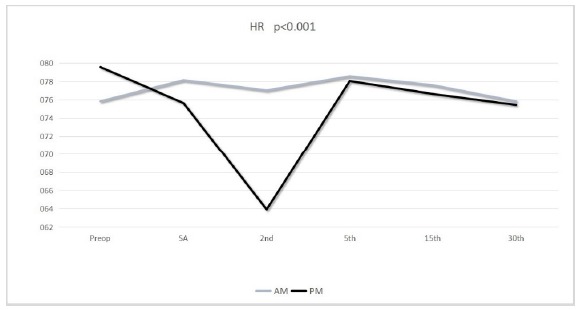
Graphical demonstration for comparison of HR values versus time between groups.

Mean preoperative heart rate of the patients was 75.8±12.6 bpm in the AM group patients and 79.6±9.5 bpm in the PM group patients. The difference between two groups was statistically significant (p<0.05). There was a significant decrease in HR versus time of PM group patients (Table 3). HR values of the PM group patients were significantly decreased at the 2^nd^ the 5th and the 15^th^ minute with regard to preoperative values compared to the AM group patients (p<0.05) (Fig. 2). 

**Table 3:** Comparison of change in percentage of HR values between groups^a^.

	AM Mean Change (%)(Min;Max)	PM Mean Change (%)(Min;Max)	p
SA-preop	3.1 (-18.2; 27.1)	-4.9 (-30.9; 20)	<0.001
2^nd^min-preop	1.9 (-29.2; 27.3)	-19.2 (-7.7; 37.4)	<0.001
5^th^min-preop	3.7 (-35.4; 33.0)	-1.5 (-36.2; 43.3)	<0.001
15^th^min-preop	2.4 (-30.8; 24.6)	-3.3 (-35.3; 35)	<0.001
30^th^min-preop	-0.2 (-46.9; 23.6)	-4.8 (-37.1; 36.4)	0,002
2^nd^min- SA	-1.1 (-12.9; 18.9)	-14.5 (-38.4; 8.3)	<0.001
5^th^min - 2^nd^ min	2.1 (-39.6; 17.4)	24.3 (-33.3; 83.6)	<0.001
15^th^ min- 5^th^ min	-1.2 (-22.2; 12.7)	-1.5 (-11.3; 37)	0.426
30^th^ min -15^th^ min	-2.0 (-56.5; 20.0)	-1.5 (-18.3; 34.2)	0.292

a. SA: right after spinal anesthesia, min:minutes, preop: preoperative measurement time.

In our study, mean Sp0_2_ of the AM group patients was 96.9±2.5% and mean Sp0_2_ for the PM group patients was 96.8±2.0%. There was no linear correlation between duration of the operation and Sp0_2_ (p>0.05). There were no statistically significant difference of Sp0_2_ changes between groups at any measurement time (p>0.05). 

**Figure 3:**
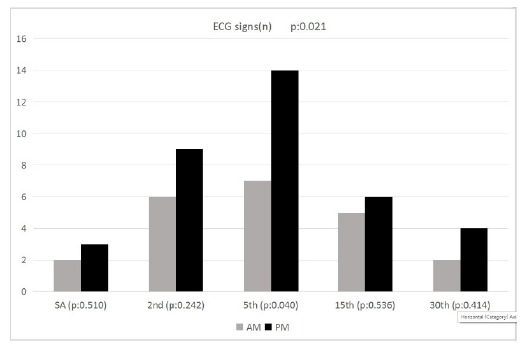
Graphical demonstration for comparison of number of ischemic ECG signs versus time between groups.

Evaluation of the ECGs in our patients showed ischemic ECG changes in 11 (9.6%) of the AM Group patients and 20 (20.8%) of the PM Group patients. There was statistically significant difference in number of ischemic ECG signs observed in the PM group patients (p=0.021). While no statistically significant difference was demonstrated between groups in measurements right after placement of SA, and at the 2^nd^, the 15^th^, and the 30^th^ minutes; number of ischemic ECG signs at the 5^th^ minute after SA was significantly higher in the PM group (p<0.05) (Fig. 3). ECG changes of 31 patients were broken down as 17 (54.8%) ST segment depression, 6 (19.4%) ST segment elevation and 8 (25.4%) negative T wave. There were no statistically significant difference between groups for the type of ECG signs (p>0.05). 

Mean body temperature of the patients was 36.6±0.3°C in the AM Group patients and 36.5±0.3°C in PM Group patients. Body temperatures of the AM group patients were significantly higher (p=0.001). 

Following SA 20 (9.5%) patients in total (5 AM/15 PM) required an administration of atropine due to bradycardia and 36 (19.5%) patients in total (13 AM/23 PM) required an administration of ephedrine due to hypotension. Atropine (p=0.005) and ephedrine (p=0.015) requirements in the PM group were significantly higher in comparison to the AM group.

## Discussion

 One of the most important preoperative preparations aimed at reducing the risk of pulmonary aspiration of gastric contents, preoperative fasting, is the restriction of oral fluid and food intake for a certain time. ASA preoperative guidelines suggest that a six hours of oral food restriction following a light meal and a two hours of fluid restriction following a clear fluid is safe for patients undergoing surgery^1^. Longer fasting periods were proposed to be the cause of various deleterious effects such as distress, fatigue, restlessness, dehydration, electrolyte imbalances and hypoglycemia^3^ ^,^ ^8^ ^,^ ^9^. In addition, hunger stimulates gastric acid secretion, both increasing gastric volume and decreasing gastric pH, thus, increasing the risk of pulmonary aspiration of gastric contents^10^. Fluid losses continue to occur during the fasting period via urine production and in the form of insensible fluid loss resulting hypovolemia^4^ ^,^ ^10^. Hypovolemia decreases tissue perfusion resulting in perioperative organ damage. 

This being the case, studies demonstrate that, in clinical settings traditional longer overnight fasting rather than evidence based shorter preoperative fasting periods still persist in many centers^4^ ^-^ ^7^. These extended fasting periods might be caused by technical insufficiencies, staff shortages, economic circumstances and unexpectedly prolonged surgery durations. De Aguilar-Nascimento et al suggested more than 80% of the patients were operated no earlier than 8 hours following cessation of oral ingestion, moreover, in 46.2 % of the cases fasting periods extend over 12 hours^4^. Another study by Bilehjani *et al.* ^5^ suggested mean preoperative fasting period to be 12.5 hours for foods and 11.5 hours for fluids. Although patients undergoing elective surgeries are prescribed a fasting period of 6 to 8 hours, our study showed that mean preoperative fasting period in our hospital was 11±3.7 hours for fluids and 13.6±3.5 hours for foods. Moreover, fasting periods of the patients operated in the afternoon hours were significantly longer in our study, with an average of 12.7 hours for liquids and 15.5 hours of fasting for foods, meaning that a strict overnight fasting was in effect in our hospital. Unfortunately, as we expected much more milder prolongations, we lacked to investigate the reasons underlying these unexpected extensions of fasting periods in this study. Most likely reasons include lack of staff to overwatch correct fasting times and to deliver oral carbohydrates preoperatively as well as the economic cost lack and of availability of these oral solutions. Inadequate informing at the preoperative visit due to a vast number of patients due operation daily might pose as another reason. 

While some studies showed preoperative fasting was not the cause of a decrease in perioperative MAP values^11^ ^,^ ^12^. Meisner *et al.* ^13^ compared different preoperative fasting periods of 6 and 12 hours and found significant difference between groups, especially during the first 18 minutes following anesthetic induction and a more frequent need for intervention in patients of the longer fasting group. Tekgul et al found that MAP values in patients undergoing surgery in the afternoon hours were significantly lower at the 2^nd^ minute in comparison to patients undergoing surgery in the morning hours, and that hypotension rates were significantly higher in patients undergoing surgery in the afternoon hours^14^. Our patients had similar MAP values preoperatively, however, a statistically significant decrease in MAP values of the PM group patients between 0-2 minutes and a statistically significant increase in the mean MAP values in the PM group patients between 2-5 minutes in comparison to AM group patients suggest that longer fasting group patients experience earlier hypotension and earlier interventions are directed towards correction of their blood pressure. A significant decrease in HR following spinal anesthetics until the 2^nd^ minute and the increase between the 2^nd^ to 5^th^ minutes accompanied the trend of MAP change in the PM group patients. 

We weren’t able to find any other studies investigating the effect of fasting on ischemic ECG signs. In our study, we found that patients in the PM group demonstrated ischemia signs on ECG more frequently. There was a statistically significant difference in the number of ischemic ECG signs at the 5^th^ minute of the operation in the PM group patients. Although temporary, these ECG signs may be a serious concern in geriatric and high risk populations. Hypotension resulting from cancellation of a higher sympathetic tone following SA might have caused a decrease in coronary artery perfusion pressure, triggering myocardial ischemia which in turn, results in the ST segment changes and/or negative T waves. Hypovolemia resulting from extended fasting might have facilitated this response. Elevated anxiety and stress response due to fasting may have contributed to the situation. Another possible reason for the ischemic findings on ECG may be the lack of electrolytes, such as; sodium and potassium. Although, extended fasting in our study might not cause this much decrease of electrolytes solely, it might have intensified already existing electrolyte imbalances. 

## Conclusıons

 Shorter preoperative fasting periods are proven to be safe by various studies. Nevertheless, a significant number of centers continue to practice longer, traditional overnight fasting. This is not only unobliging; but also harmful to patients. Our study has shown that fasting times in our population is far longer than recommended and fasting prolonged more than 15 hours is related to a transiently increased cardiac stress and mild hypothermia. Yet, bringing the valid guidelines in effect might prove difficult and costly in busy tertiary centers, such as our hospital. Forming perioperative nutrition teams to overwatch patients’ nutritional status, especially in high risk populations, such as geriatrics, could improve the quality of healthcare. 
